# Dynamic Calcium Release From Endoplasmic Reticulum Mediated by Ryanodine Receptor 3 Is Crucial for Oligodendroglial Differentiation

**DOI:** 10.3389/fnmol.2018.00162

**Published:** 2018-05-18

**Authors:** Tao Li, Lingyun Wang, Teng Ma, Shouyu Wang, Jianqin Niu, Hongli Li, Lan Xiao

**Affiliations:** Chongqing Key Laboratory of Neurobiology, Department of Histology and Embryology, Third Military Medical University, Chongqing, China

**Keywords:** oligodendrocyte progenitor cells (OPCs), oligodendrocytes (OLs), ryanodine receptor 3 (RyR3), Ca^2+^ release, differentiation, caffeine

## Abstract

Increased intracellular Ca^2+^ in oligodendrocyte progenitor cells (OPCs) is important to initiate their differentiation, but the intracellular Ca^2+^ channel involved in this process remains unclear. As a Ca^2+^-induced Ca^2+^ release (CICR) channel that mediates endoplasmic reticulum (ER) Ca^2+^ release, the role of ryanodine receptors (RyRs) in oligodendroglial development is unexplored. In the present study, we observed that among the three mammalian isoforms, oligodendroglial lineage cells selectively expressed RyR3. Strong RyR3-positive signal was distributed all over the cytoplasm and processes in OPCs and/or immature OLs (imOLs), whereas it gradually decreased and was located mainly around the perinuclear region in mature oligodendrocytes (OLs). In addition, RyR3-mediated intracellular Ca^2+^ waves following caffeine stimulation were correlated with the expression pattern of RyR3, in which high flat Ca^2+^ fluctuations and oscillatory Ca^2+^ waves were more frequently recorded in OPCs and/or imOLs than in OLs. Through further functional exploration, we demonstrated that pretreatment with the RyR antagonist ryanodine could neutralize the increase in intracellular Ca^2+^ induced by OPC differentiation and reduce the number of mature OLs. Moreover, gene-level knockdown of RyR3 by lentivirus in OPCs resulted in inhibition of OPC differentiation. Taken together, our results provide new insight into the crucial role of RyR3-mediated ER Ca^2+^ release in the regulation of OPC differentiation and/or myelination.

## Introduction

In the CNS, myelinating oligodendrocytes (OLs) originate from oligodendrocyte progenitor cells (OPCs) after passing through a series of distinct developmental stages, i.e., OPCs, immature OLs (imOLs) and mature OLs (Stangel and Hartung, 2002). As impairment of OL differentiation has been considered to be the major cause of remyelination failure, which occurs in numerous demyelination diseases such as multiple sclerosis (MS; Wolswijk, 1998; Chang et al., 2002; Kuhlmann et al., 2008), promoting OPC differentiation into mature OLs becomes a promising approach for myelin repair. However, the mechanism regulating oligodendroglial differentiation remains to be elucidated. As a critical functional pattern of non-excitable glia cells, Ca^2+^ signaling is essential for oligodendroglial differentiation and myelination (Kirischuk et al., 1995; Cohen et al., 1996; Yoo et al., 1999; Soliven, 2001; Fulton et al., 2010; Cheli et al., 2015; Friess et al., 2016; Baraban et al., 2018; Krasnow et al., 2018). For instance, inhibition of the voltage-operated Ca^2+^ entry in OPCs repressed their maturation and the myelin forming ability (Cheli et al., 2015). Increasing resting intracellular Ca^2+^ through membrane depolarization could facilitate MBP synthesis in OPCs (Friess et al., 2016). Newest studies further provided *in vivo* data showing that the Ca^2+^ transients in OLs could regulate retraction and elongation of the developing myelin sheath (Baraban et al., 2018; Krasnow et al., 2018). However, the Ca^2+^ channels involved in oligodendroglial differentiation is believed to be important but remains largely unexplored.

It is known that endoplasmic reticulum (ER) is the major intracellular Ca^2+^ pool (Meldolesi and Pozzan, 1998) and that ER Ca^2+^ release is driven mainly by inositol-1,4,5,-trisphosphate receptors (IP3Rs) and ryanodine receptors (RyRs; Koulen and Thrower, 2001). In OPCs, both IP3R2 and ryanodine receptor 3 (RyR3) can mediate highly localized Ca^2+^ release, of the types called “puffs” and “sparks”, respectively (Haak et al., 2001), but only IP3R2 is able to initiate Ca^2+^ waves under pharmacological treatments (Haak et al., 2001). However, the functions of those channels during oligodendroglial development remain unclear. Series of studies demonstrate that, compared with IP3Rs, the opening of which requires both Ca^2+^ and IP3 (Moraru et al., 1999; Foskett et al., 2007), RyRs are Ca^2+^-induced Ca^2+^ release (CICR) channels that can be triggered merely by a low concentration of Ca^2+^ (~1 μM; Meissner et al., 1986, 1997; Bezprozvanny et al., 1991), and this CICR function has been shown to powerfully amplify small inward Ca^2+^ currents in NG2 glial cells (Haberlandt et al., 2011). Therefore, we propose that RyR3 is likely a critical bridge for the formation of intracellular Ca^2+^ signaling and thus participates in the regulation of oligodendroglial development.

In the present study, we sought to characterize the expression and function of RyRs during OPC differentiation. Our results showed that RyR3 was selectively expressed and widely distributed in the soma and processes of the oligodendroglial lineage cells and that its expression level was downregulated following OPC differentiation. Using confocal Ca^2+^ imaging, we found that the ER Ca^2+^ release after caffeine stimulation was much stronger in OPCs and imOLs than in mature OLs. Moreover, inhibiting the function of RyR3 either pharmacologically or by gene knockdown suppressed the differentiation of OPCs. Our results revealed a critical role for RyR3-mediated Ca^2+^ signaling in oligodendroglial differentiation that may provide new insight into therapeutic approaches for demyelinating diseases.

## Materials and Methods

### OPC Culture

Cortical OPCs were purified as previously described (Niu et al., 2012b). Briefly, the mixed glial cells were isolated from cortex of postnatal day 1–3 neonatal Sprague-Dawley (SD) rats and enriched in OPC growth medium followed by two passages to enrich cell numbers. OPC proliferation medium was DMEM/F12 + 1% N2 supplement + PDGFAA. OPCs were induced to differentiate by replacing the medium with OPC differentiation medium: DMEM/F12 + 1% N2 supplement + 5 mg/mL N-acetyl-L-cysteine (Amresco) + 1% fetal bovine serum + 5 mg/mL insulin.

Reagents used were as follows: Dulbecco’s modified Eagle’s medium/F12 (DMEM/F12; Hyclone, SH30023), N2 supplement (Invitrogen, 17502048), fetal bovine serum (FBS; Hyclone, SV30087), insulin (Sigma, I6634), N-acetyl-l-cysteine (NAC; AMRESCO, 0LA0011), PDGFAA (Peprotech, 100-13A). The SD rats related procedures were performed in accordance with the guidelines approved by the Laboratory Animal Welfare and Ethics Committee of the Third Military Medical University (Niu et al., 2016).

### Confocal Ca^2+^ Imaging Measurements

OPCs were grown and differentiated in glass-bottom dish and loaded with the fluorescent Ca^2+^ sensitive dye Fluo-3AM (5 μM, Invitrogen) for 20 min at 37°C in a modified imaging buffer containing (in mM): NaCl, 135; KCl, 3; MgCl_2_, 2; Glucose, 8; HEPES, 10 and CaCl_2_, 2 (pH adjusted to 7.4 with NaOH). The dye-loaded cells were washed twice and maintained for at least 20 min at RT in fresh imaging buffer to allow complete dye de-esterification. Ca^2+^ wave and fluorescence images were real-time recorded for at least 15 min in all experiments, using a confocal laser-scanning microscope (FluoView FV1000, Olympus, Japan) with the UplanFl40× objective (N.A. 0.95). The image acquisition frequency is 100–180 ms/image. Cell morphology was detected using differential interference contrast (DIC) under confocal microscopy. Ca^2+^ concentrations were measured by exciting Fluo-3 AM at 488 nm.

Intracellular Ca^2+^ responses in OPCs, imOLs and mature OLs were recorded with caffeine (20 mM, Sigma, Ca^2+^ free) stimulation in a Ca^2+^ free imaging buffer. For spontaneous Ca^2+^ recordings, OPCs were grown in proliferation medium or differentiation medium for 6 h with or without ryanodine treatment (50 μM, TOCRIS, 1329, 10 min). Fluo-3 loading and cell washing was followed by applying normal proliferating medium (Pro-medium) or differentiating medium (Diff-medium) for the following recording.

### Cell Processing and Immunocytochemistry

Cells were grown on coverslips, fixed in cold 4% paraformaldehyde for 20 min, rinsed with 0.01 M PBS, blocked with 1% bovine serum albumin (BSA) and 0.2% Triton-X100 for 30 min and then incubated with primary antibodies diluted in 1% BSA overnight at 4°C and then by fluorophore-conjugated secondary antibodies at room temperature (RT) for 2 h. Cell nuclei were stained with 4′,6-diamidino-2-phenylindole (DAPI, Thermo Fisher) for 10 min.

In this study, the following antibodies were used: mouse polyclonal anti-Olig2 (1:500, Millipore, MABN50), rabbit polyclonal anti-RyR3 (1:200, Millipore, AB9082), and goat anti-myelin basic protein (MBP; 1:500, Santa Cruz, sc13914). The secondary antibodies were as follows: Alexa 568-labeled donkey anti-mouse (1:1000, Invitrogen), Alexa 488-labeled donkey anti-goat (1:1000, Invitrogen), Alexa 568-labeled donkey anti-goat (1:1000, Invitrogen), Alexa 568-labeled donkey anti-rabbit (1:1000, Invitrogen) and Cy5-labeled rabbit anti-mouse (1:500, Jackson ImmunoResearch).

### Image Acquisition and Quantification

Fluorescent images were captured using an Axio Imager M2 fluorescence microscope (Zeiss, Oberkochen, Germany) or a confocal laser-scanning microscope (Olympus, IV 1000, Shinjuku, Tokyo) with excitation wavelengths appropriate for Alexa Fluor 488 (488 nm), 596 (568 nm), 647 (628 nm) or DAPI (380 nm). Digital images of the oligodendroglial lineage cells in the supplemental figure were acquired with an Olympus IX51 microscope with an Olympus C-7070 camera (Tokyo). For the statistical analysis, randomly selected images in at least three representative fields were acquired from each sample. Detection and quantification were performed using ImageJ software (National Institutes of Health, NIH).

### Western Blot Analysis

The cells were lysed using RIPA lysis buffer (Beyotime, P0013B) with freshly added 1% phenylmethylsulfonyl fluoride (PMSF, Amresco, O754) solution. Protein concentration was determined using Coomassie brilliant Blue G-250. SDS-PAGE and Western blotting were carried out as reported previously (Niu et al., 2012a). Proteins were transferred to polyvinylidene difluoride (PVDF) membranes and visualized by chemiluminescence (ECL Plus, GE Healthcare, Marlborough, MA, USA) after incubation with the antibodies. β-actin was used as the loading control. Quantification of band intensity was performed using ImageJ software. The primary antibodies included the following: rabbit polyclonal anti-RyR3 (1:1000, Millipore), rabbit polyclonal anti-platelet-derived growth factor receptor α (PDGFRα; 1:1000, Santa Cruz, sc-338), mouse anti-2′,3′-cyclic nucleotide-3′-phosphodiesterase (CNPase; 1:1000, Abcam, ab6319), goat anti-MBP (1:1000, Santa Cruz) and mouse anti-β-actin (1:2000, Santa Cruz, sc-47778). The secondary antibodies included the following: goat anti-mouse-HRP (1:2000, Santa Cruz, sc-2094), goat anti-rabbit-HRP (1:2000, Santa Cruz, sc-2313) and rabbit anti-goat-HRP (1:2000, Santa Cruz, sc-2020).

### RT-PCR Analysis

Total ribonucleic acid (RNA) was isolated from different stages of OPC cultures (OPC differentiated for 1 day, 2 days, or 4 days using TRIzol (Life Technologies). Real-time polymerase chain reaction (RT-PCR) was performed with the C1000 Touch™ Real-time PCR Detection System (Bio-Rad) and GoTaq^®^ qPCR Master Mix (Promega, Sunnyvale, CA, USA). The amplification procedure and melt curve analysis were performed using three independent replicates for each sample.

The oligonucleotide primers used were as follows:

**Table:** 

	Forward	Reverse	Tm
RyR1	GAGGGTGATGAAGATGAGAAC	TCCCGCCCGAAGATGTC	60°C
RyR2	GCTGGCCCTGTTTGTTG	ATCCATGCCCAGTAACTCGCT	61.3°C
RyR3	CTGTGTGGTGGGCTATTACTG	TGCTTTGGCCTCTTCTACTG	58.3°C
MBP	GAGACCCTCACAGCGACAC	ATCCAGAGCGGCTGTCTC	59°C
β-actin	CGTTGTACATCCGTAAAGACC	CATCGCACTCCTGCTTGCT	58°C

### Lentivirus Mediated shRNA Interference

The shRNA lentivirus was purchased from Obio Technology, Shanghai. Targeted sequence in rat RyR3 gene is: AGATGCTAATTGCATCTC. The primary lentivirus solution was diluted in several gradient concentrations (1:10, 1:100, 1:300, 1:500, 1:1000) to check the best work concentration (normal oligodendroglial viability and good interference efficiency). The lentivirus was diluted 1:300 in the differentiation medium with a primary concentration of 8.67*10^8^ transducing units (TU)/ml for the interference group (shRyR3) and 6.71*10^8^ TU/ml for the control group (shCTL). Lentivirus was removed after transfection for 16 h. OPC were further differentiated for another 32 h and fixed.

### Antisense Oligonucleotides

Phosphodiester ODNs protected by terminal phosphorothioate double substitution (capped ODNs) against possible exonuclease-mediated degradation were purchased from Tib-Molbiol (Sigma). The sequences are as follows: anti-RyR3: 5′-A*G*ATGCTAATTGCATC*T*C-3′ (*indicates the phosphorothioate residues) and an 18-mer fully degenerated ODN (dODN), 5′-N*N*NNNNNNNNNNNNNN*N*N-3′ (where N is G, C, A, or T), which was used as a control ODN. ODNs were transported using an artificial cationic lipid (DOTAP; Sigma) to enhance both uptake and stability. Antisense ODNs (aODNs) or dODNs were pre-incubated at 37°C for 30 min. Differentiated OLs were collected on day 3.

### Statistics Analysis

All experiments were repeated at least three times. Data are shown as the means ± SEM. Statistical analyses of three groups were performed using one-way analysis of variance (ANOVA) followed by Tukey’s *post hoc* test. Comparisons between two experimental groups were made using Student’s *t*-test (GraphPad Prism 6). A probability of *p* < 0.05 was considered significant.

## Results

### Oligodendrocytes Selectively Express RyR3, Which Is Downregulated During Differentiation

Similar to our previous studies (Niu et al., 2012b), different stages of oligodendroglial cells in our culture system were identified based on morphological features using DIC and immunostaining for stage-specific markers. Normally, OPCs have small, round cell bodies with a bipolar morphology (Supplementary Figure S1). After 2 days in the differentiation medium, imOLs with 3–5 primary processes and a sparse arborization predominate this stage (Supplementary Figure S1). After 4 days of differentiation, mainly mature OLs characterized by multipolar processes and a rich arborization were observed (Supplementary Figure S1). These features indicate the accuracy of our culture model for studying the developmental schedule of the OL lineage.

**Figure 1:**
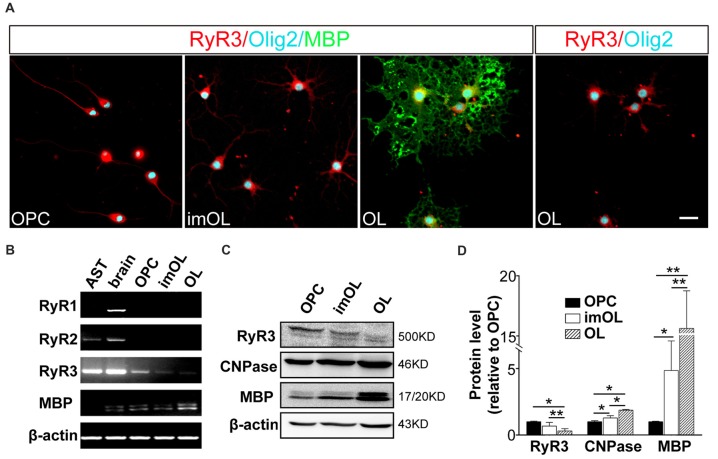
Oligodendroglial cells selectively express ryanodine receptor 3 (RyR3), which is downregulated during differentiation. **(A)** Immunofluorescence staining of RyR3, Olig2 (oligodendroglial lineage marker) and myelin basic protein (MBP; mature OL marker) in three stages of cultured OL lineage cells. RyR3 was strongly distributed all over the cytoplasm and processes in oligodendrocyte progenitor cells (OPCs) and immature oligodendrocytes (imOLs), while in mature OLs, RyR3 signal was mainly located in the cell body and showed lower expression in the primary processes. Scale bar 20 μm. **(B)** mRNA levels of RyRs in OL lineage cells, astrocytes (AST) and rat brain tissue. **(C,D)** Western blot analysis showed increased levels of myelin proteins (CNPase, MBP) and decreased levels of RyR3 protein during OPC development (*p < 0.05, **p < 0.01).

To determine the RyR subtypes expressed in OLs, we first analyzed the mRNA levels in OL lineage cells. We found that only RyR3, the known “brain type”, was detected in OL lineage cells. In parallel experiments, RyR3 mRNA was also found in astrocytes, RyR2 mRNA was found in astrocytes and rat brain tissue, and RyR1 mRNA was only found in brain tissue (Figure 1B). Interestingly, the RyR3 mRNA level gradually decreased during OPC differentiation, and this downregulation tendency was further confirmed by western blot analysis (Figures 1C,D).

Next, we clarified the RyR3 distribution pattern in OL lineage cells by immunostaining. It has been shown that RyR3 is the subtype of ryanodine receptors expressed in OPCs. Specifically, RyR3 is located throughout the cell body and processes of OPCs, except in the region of the nuclear membrane (Haak et al., 2001). Our present results also revealed the enrichment of RyR3 in the processes and non-nuclear area of OPCs and imOLs, while in mature OLs, RyR3 was mainly located in the cell body and primary processes (Figure 1A).

Taken together, these results reveal a spatiotemporal regulated expression pattern of RyR3 in OL lineage cells, indicating its potential in regulating OL development.

### ER Ca^2+^ Release Following Caffeine Stimulation Is Stage Specific During OPC Differentiation

**Figure 2:**
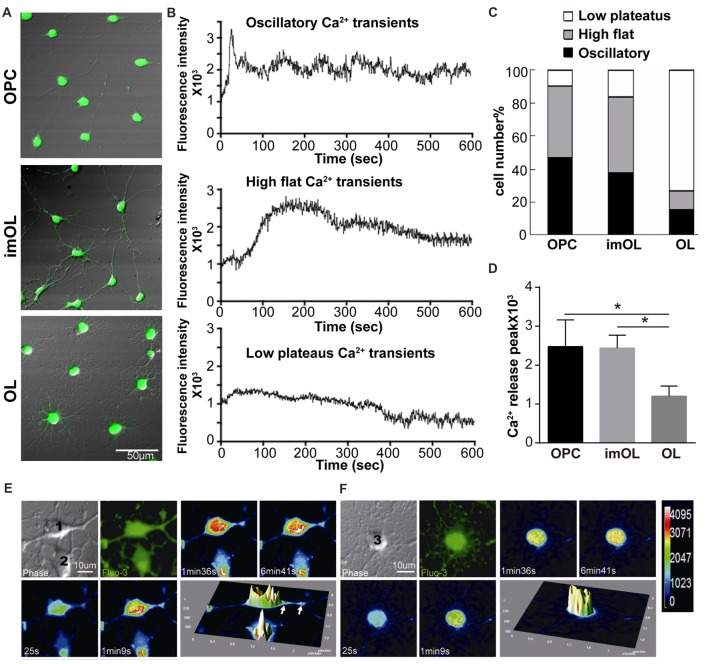
Endoplasmic reticulum (ER) Ca^2+^ release following caffeine stimulation is stage specific during OPC differentiation. **(A)** Differential interference contrast (DIC) with Fluo-3 fluorescence of three stages of OL lineage cells, showing typical shape of OPC, imOL and OL. Note that the pictures were acquired at the end point of Ca^2+^ imaging to ensure the morphology of the oligodendroglial cells, thus Fluo-3 fluorescence was extremely strong. **(B)** Representative examples of Ca^2+^ response following caffeine stimulation. Note that the start point in the x-axis is not the beginning point of recording, baseline recording is omitted from the curve. **(C)** OPCs and imOLs, which tend to respond with oscillatory Ca^2+^ transients and high flat Ca^2+^ transients, reacted stronger than OLs, which tend to respond with low plateaus Ca^2+^ transients. **(D)** OPC and imOL had significantly higher Ca^2+^ release peaks after caffeine stimulation compared with mature OLs (*p < 0.05). Time-lapse Ca^2+^ imaging induced by caffeine (20 mM, 0 Ca^2+^) in an OPC (1) **(E)** and a mature OL **(F)**. Four successive scans were selected from a series of images obtained within 10 min. Transient fluorescence fluctuations, representing local Ca^2+^ release events, were indicated by the pseudocolored peaks in the OPC cell body and processes (arrows), but they occurred only in the soma in mature OLs. The pseudocolor bar in **(F)** showed the fluorescence intensity of different colors.

Although OPCs are reported to have spontaneous oscillatory-like Ca^2+^ activity with peak and plateau transients, while mature OLs show “flat” Ca^2+^ signaling (Niu et al., 2016), the ER Ca^2+^ channels in OPCs (RyR3 and IP_3_R2) are relatively quiescent in comparison to those in neurons (Haak et al., 2001). To better study the RyR channel function, we took advantage of the classical RyR agonist caffeine (20 mM, 0 Ca^2+^; Zucchi and Ronca-Testoni, 1997) and real-time recorded the intracellular Ca^2+^ concentration with confocal microscopy. The Ca^2+^ release peaks are significantly higher in OPC and imOLs than in mature OLs (Figure 2D). More importantly, three typical Ca^2+^ responses were recorded in OL lineage cells after caffeine stimulation, showing stage-specific characteristics (Figures 2A,B). The high flat Ca^2+^ response following caffeine application was mainly found in OPCs (53.7%, *n* = 65) and imOLs (54.8%, *n* = 72). Likely, the oscillatory Ca^2+^ transients were present at a higher ratio in OPCs (35.5%, *n* = 65) and imOLs (27.9%, *n* = 72) than in mature OLs (10.5%, *n* = 76). The low plateaus Ca^2+^ response was the dominant reaction of mature OLs (76.3%, *n* = 76) but was barely found in OPCs and imOLs (Figure 2C). Notably, the millimolar concentration of caffeine was previously demonstrated to be an inhibitor of the IP3R channel in glioblastoma (1–10 mM; Kang et al., 2010), cerebellar microsomes (10–50 mM; Brown et al., 1992), B lymphocytes (1–25 mM) and other cells (Sei et al., 2001). In our present study, while the corresponding Ca^2+^ influx was abolished by using a Ca^2+^-free extracellular solution, the caffeine (20 mM)-induced Ca^2+^ transient is likely due to release of Ca^2+^ from ER mediated mainly by RyR3 channel. We assume that this stage-specific response is probably correlated to the downregulated expression of RyR3 during OL development.

Given that RyR3 is not homogeneously distributed in the cell body and cell processes (Haak et al., 2001), especially in mature OLs, as we showed in Figure 1A, we wonder whether there is a regional difference in the Ca^2+^ response. By transforming the time-lapse Ca^2+^ imaging into pseudocolor changes and 3D surface plots with Image J software, we found that OPCs respond rapidly and strongly in both the soma and processes (Figure 2E), whereas only somal regions showed a slow and weak Ca^2+^ response in mature OLs (Figure 2F). Thus, the Ca^2+^ response pattern is highly correlated with the expression level and distribution of RyR3 in OL lineage cells.

At this point, we clarified the spatiotemporal regulated expression pattern of RyR3 and its expression-correlated functional pattern after caffeine stimulation. Our results indicate that RyR3-mediated Ca^2+^ signaling actively participates in the developmental regulation of OL lineage cells. The role of RyR3 under physiological conditions requires further exploration.

### Inhibition of RyR3 Function by Ryanodine Suppresses OPC Differentiation

**Figure 3:**
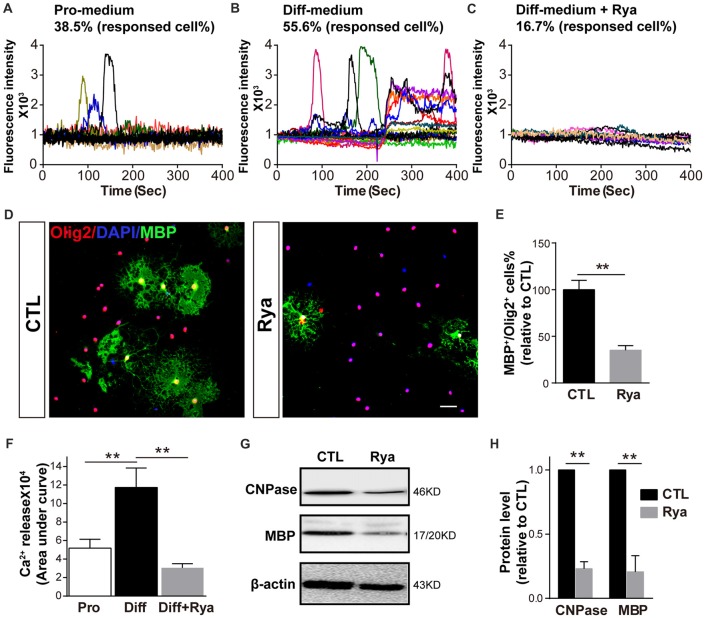
Inhibition of RyR3 by ryanodine suppresses OPC differentiation. **(A)** Ca^2+^ recording of OPCs in proliferation medium (Pro-medium) showing random spontaneous waves. **(B)** OPCs cultured in differentiation medium (Diff-medium) for 6 h showed frequently higher peak waves, which could be inhibited by ryanodine (Rya) **(C)**. **(F)** The total amount of Ca^2+^ release, reflected by the area under curve, was greater in early differentiating OPCs than in proliferating OPCs (**p < 0.05), and the release activity in early differentiating OPCs was greatly reduced by ryanodine (**p < 0.05). **(D,E)** There were fewer MBP-positive mature OLs after treatment with ryanodine (50 μM) for 72 h. Scale bar 50 μm. **(G,H)** Western blot analysis showed the same inhibitory effect of ryanodine on OPC differentiation (**p < 0.05).

As Ca^2+^ signaling is essential for OPC differentiation and myelination, especially the initiation of OPC differentiation (Cheli et al., 2015; Friess et al., 2016), we next investigated whether there is active Ca^2+^ signaling at the initial stage of OPC differentiation. Spontaneous Ca^2+^ signaling was recorded in OPCs cultured in the proliferation medium and in OPCs that had been cultured in differentiation medium for 6 h. Consistent with our previous research (Niu et al., 2016), approximately 38.5% of OPCs presented occasional Ca^2+^ elevation (*n* = 18), which was characterized by a gradual increase in the Ca^2+^ concentration to a peak and a subsequently decreasing Ca^2+^ signal (Figure 3A). Meanwhile, in OPCs that were induced to initiate differentiation, spontaneous Ca^2+^ activity was observed in 55.6% of cells (*n* = 20). Those Ca^2+^ activities appeared at higher frequency; moreover, the elevation of Ca^2+^ signaling was more persistent in certain individual cells (Figure 3B). It has been reported that ryanodine, a RyR-specific blocker, locks the RyR channel into a “closed state” at higher concentrations (>50 μM; Meissner, 1986; Lai et al., 1989; McGrew et al., 1989). Thus, we used 50 μM ryanodine as an antagonist to block RyR3. The differentiation-related Ca^2+^ activity was dramatically inhibited (*n* = 24; Figures 3C,F). The amount of Ca^2+^ release reflected as the area under curve was markedly greater in early differentiating OPCs than in proliferating OPCs (Figure 3F). This result indicates that RyR3-mediated Ca^2+^ release from the ER is a critical process during OPC differentiation. To further verify the function of RyR3 in oligodendroglial differentiation, we treated OPCs with ryanodine (50 μM) and measured the number of mature OLs by immunofluorescence staining after 3 days of differentiation. The number of MBP-positive OLs was significantly decreased in the ryanodine-treated group (Figures 3D,E). Importantly, cell viabilities of OPC cultures were not affected by ryanodine treatment as reflected by the nuclear number, which is consistent with previous works (Matyash et al., 2002; Ruiz et al., 2010). Western blot results showed decreased protein levels of MBP and CNPase, which also reflected the inhibition of oligodendroglial maturation (Figures 3G,H). Thus, our results demonstrate that RyR3-mediated Ca^2+^ signaling does participate in the differentiation of OPCs.

### Knockdown of RyR3 in OPCs Results in Inhibition of OPC Differentiation

**Figure 4:**
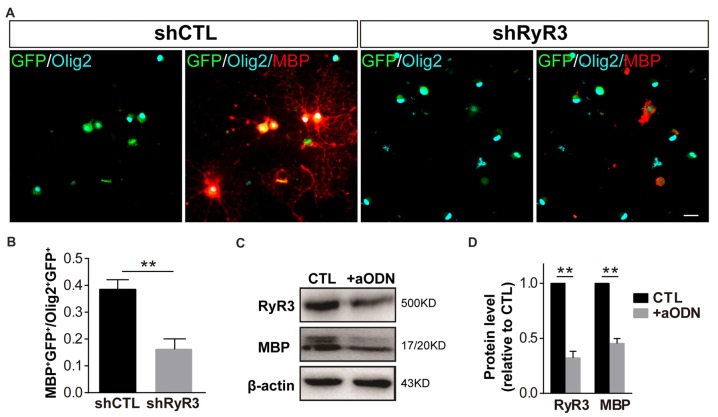
Knockdown of RyR3 in OPCs results in inhibition of OPC differentiation. **(A,B)** Gene knockdown of RyR3 with shRNA interference mediated by lentivirus infection showed fewer MBP-positive mature OLs after 48 h of differentiation. GFP was expressed in lentivirus-infected cells (**p < 0.05). Scale bar 20 μm. **(C,D)** Knockdown of RyR3 in OPCs by antisense oligonucleotide (+aODN) resulted in less expression of myelin basic protein (MBP; **p < 0.05).

To further detect the role of RyR3 in OPC differentiation, we performed lentivirus-mediated gene knockdown of RyR3, which more precisely targets RyR3. OPCs were induced to differentiate after infection with an RNA interference lentivirus (shRyR3) or a control lentivirus (shCTL). After differentiation for 48 h, we observed efficient infection of both lentiviruses, visualized as the GFP expression in the cytoplasm (Figure 4A). Immunofluorescence staining for Olig2 (OL lineage marker) and MBP (mature OL marker) showed that the percentage of MBP-positive OLs was significantly decreased and most of the MPB-positive cells showed smaller process areas, and less flat membrane structures after RyR3 knockdown (Figures 4A,B), indicating the blockage of OPC differentiation in the absence of RyR3-mediated Ca^2+^ signaling. Cell viability after lentivirus treatment was guaranteed by the unchanged Olig2 positive cell number. As antisense oligonucleotides (aODNs) have the ability to selectively reduce the mRNA level of RyR3 (Galeotti et al., 2008), we also applied aODNs in OPC cultures and confirmed that aODNs reduced the protein level of RyR3. Consistent with the shRNA effect, aODN treatment similarly reduced the number of mature OLs (data not shown) and the levels of MBP (Figures 4C,D). Taken together, these observations reveal that gene-level knockdown of RyR3 induces loss of RyR3 channel function in OPCs, finally resulting in inhibition of OPC differentiation, implying that RyR3-mediated Ca^2+^ signaling plays an essential role during OPC differentiation.

## Discussion

**Figure 5:**
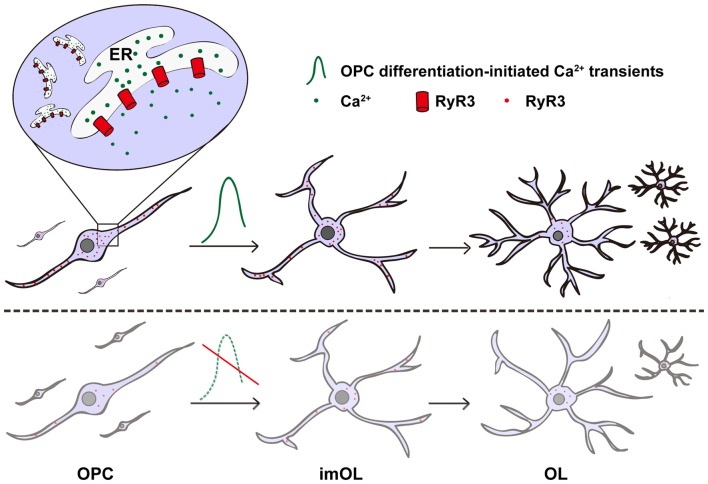
Scheme illustrating the contribution of RyR3-mediated Ca^2+^ signaling during oligodendroglial development. RyR3, a Ca^2+^-induced Ca^2+^ release (CICR) channel located on the ER membrane during oligodendroglial differentiation, note that it is widely distributed in the cell body and processes of OPC/imOLs but down-regulated and restricted in the cell body of mature OLs. The initiation of OPC differentiation requires hyperactive Ca^2+^ signaling mediated by RyR3. RyR3 knockdown or blockage of RyR3-mediated Ca^2+^ signaling in OPCs results in inhibition of OPC differentiation.

In non-excitable oligodendroglial cells, how intracellular Ca^2+^ signaling is regulated and how it contributes to oligodendroglial differentiation remains unclear. In our present study, we systematically analyzed the expression and function of the ER Ca^2+^ release channel—RyR3. We found that RyR3 is the only RyR expressed in oligodendroglial cells and dynamically regulated during OPC differentiation. Importantly, inhibition of RyR3 resulted in blockage of OPC differentiation (Figure 5). Our results not only demonstrate the essential role of RyR3-mediated Ca^2+^ signaling for oligodendroglial differentiation but also improve our understanding of the intracellular Ca^2+^ channel function in oligodendroglial lineage cells, which has barely been studied before.

RyRs (RyR1–3) are major Ca^2+^ channels responsible for ER Ca^2+^ release, and RyR-mediated transient increase and oscillations of intracellular Ca^2+^ has been considered to be particularly important for cell functions (Zalk et al., 2007; Fulton et al., 2010; Suzuki et al., 2012). In oligodendroglial lineage cells, the RyR functionality was suggested to correlate with their developmental stages both *in vivo* and *in vitro* (Simpson et al., 1998), but the isoform of RyRs involved in this process has not been identified. Among RyRs, RyR3 is abundantly distributed in the CNS, and it functions in the activation and migration of astrocytes (Fill and Copello, 2002; Matyash et al., 2002; Galeotti et al., 2008; Lanner et al., 2010). In oligodendroglial lineage cells, only RyR3 was selectively expressed in cultured rat cortex OPCs (Haak et al., 2001), while another study demonstrated that three isoforms of RyRs (RyR1–3) were expressed in cultured rat optic nerve-derived mature OLs (Ruiz et al., 2010). Here, using rat cortex-derived OPC cultures, we confirmed the selective expression of RyR3 in oligodendroglial lineage cells and further determined the spatiotemporal expression pattern of RyR3 during oligodendroglial differentiation. Additionally, we found that enrichment of RyR3 in OPCs and imOLs corresponded to the stronger Ca^2+^ responses in those cell types than in mature OLs following caffeine stimulation. Interestingly, a previous study in myotubes has revealed that embryonic myotubes, which express RyR3, have considerably more variability in the size and kinetics of their Ca^2+^ sparks than do adult cells, which lose RyR3 expression (Ward et al., 2000). Therefore, expression of RyR3 in OPCs and imOLs likely enabled them to be more hyperactive in terms of Ca^2+^ transients and thus program them into the differentiating state.

Previous studies showed that Ca^2+^ responses to depolarization in OPCs were more active than those in mature OLs, and this phenomenon was mainly explained by the downregulation of L-type voltage-operated Ca^2+^ channels (VOCCs) following OPC maturation (Berger et al., 1992; Takeda et al., 1995; Paez et al., 2010; Zhang et al., 2014). Considering that RyR-mediated CICR is a critical amplification point for depolarization-induced intracellular Ca^2+^ elevation (Verkhratsky and Petersen, 2002; Pouvreau et al., 2007; Haberlandt et al., 2011), our results suggested that RyR3-mediated Ca^2+^ release from the ER is also an important contributor to the dynamic intracellular responses during OPC differentiation.

Consistent with a previous study (Simpson et al., 1998), we found that RyR3 was enriched in the entire processes of OPCs and imOLs but was absent in the leading processes of mature OLs, indicating that RyR3-mediated local Ca^2+^ signaling may contribute to enabling the process elongation and/or movements of OPCs (Paez et al., 2007) and to initiating cell differentiation (Friess et al., 2016). In agreement with this, we provide evidence showing that RyR3 knockdown results in failure of oligodendroglial differentiation, indicating that CICR may critically contribute to OPC differentiation.

Even if it has not been studied in this work, we speculate that in physiological *in vivo* condition, the Ca^2+^ signals triggering the opening of RyR3 are likely originated from the nearby neurons. Large amount of evidences have shown that neuronal action potentials could regulate myelin development, the candidate mediators are the neurotransmitters (glutamate, ATP, adenosine…) which could trigger Ca^2+^ influx in oligodendroglial cells and possibly evoke the following CICR through RyR3 (Butt, 2006; Spitzer et al., 2016; Sun et al., 2016; Krasnow et al., 2018). Moreover, axonal activity-induced increases in extracellular K^+^ are sufficient to depolarize VOCCs in nearby oligodendroglial cells to produce a significant rise in intracellular Ca^2+^ which also need CICR to form the final Ca^2+^ signal (Cheli et al., 2015). Importantly, RyR3 and IP3R2 are usually co-localized in OPCs, and interactions between them determine the spatial and temporal characteristics of Ca^2+^ signaling (Haak et al., 2001). Although a relative higher concentration of caffeine that was applied in our current study has been shown to stimulate RyR but inhibit the IP3R Ca^2+^ channel in several cell types (Brown et al., 1992; Sei et al., 2001; Kang et al., 2010), we could not exclude the role of IP3R2 in the Ca^2+^ signaling formation in oligodendroglial differentiation. Channel-specific antagonists or a genetic approach may be needed in our future studies.

In summary, we provide direct evidence showing that RyR3-mediated ER Ca^2+^ release is dynamically regulated and plays an essential role in initiating OPC differentiation (Figure 5). Our results supplement the current understanding of the function of intracellular Ca^2+^ channels in oligodendroglia and may provide valuable insights into therapeutic strategies for demyelinating diseases.

## Author Contributions

LX, HL and TL designed experiments and wrote the manuscript. TL, LW, TM and SW conducted the experiments. TL, LW and JN collected and analyzed the data.

## Conflict of Interest Statement

The authors declare that the research was conducted in the absence of any commercial or financial relationships that could be construed as a potential conflict of interest.

## Abbreviations

CICR: Ca^2+^-induced Ca^2+^ release
CNPase: 2′,3′-cyclic nucleotide-3′-phosphodiesterase
ER: endoplasmic reticulum
imOL: immature oligodendrocyte
MBP: myelin basic protein
OL: oligodendrocyte
OPC: oligodendrocyte progenitor cell
PDGFRα: platelet-derived growth factor receptor α
RyR: ryanodine receptor
VOCC: voltage-operated Ca^2+^ channel.
