# Supplementary material for: Dynamic Calcium Release From Endoplasmic Reticulum Mediated by Ryanodine Receptor 3 Is Crucial for Oligodendroglial Differentiation
Source: Front Mol Neurosci. 2018 May 18;11:162. doi: 10.3389/fnmol.2018.00162 (PMC5968115; doi:10.3389/fnmol.2018.00162)
Supplement: Supplementary file 1 [file Data_Sheet_1.DOC]

Supplementary Material

# Dynamic Calcium Release from Endoplasmic Reticulum Mediated by Ryanodine Receptor 3 is Crucial for Oligodendroglial Differentiation

Tao Li, Lingyun Wang, Teng Ma, Shouyu Wang, Jianqin Niu, Hongli Li*, Lan Xiao*

*** Correspondence:**

Hongli Li: [lihongli@tmmu.edu.cn](mailto:lihongli@tmmu.edu.cn)

**Lan Xiao:** [**xiaolan35@hotmail.com**](mailto:xiaolan35@hotmail.com)

**Supplementary Figure 1**


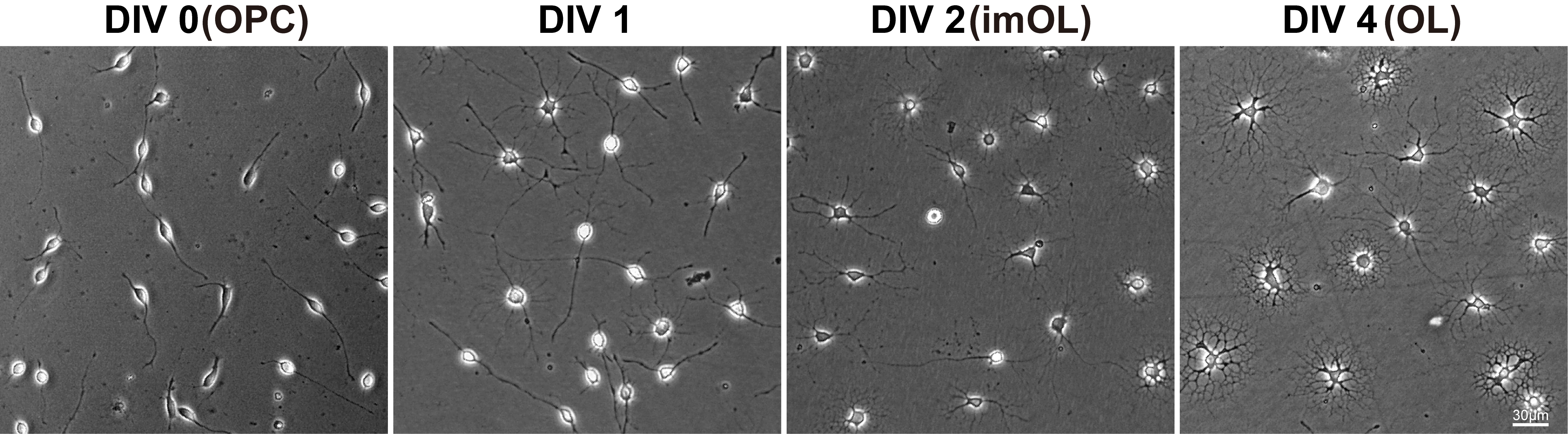


**Supplementary Figure 1.** Primary OPC culture model setup. DIC showed OPC with a small and round cell body and two processes. After 1 day of differentiation (DIV 1), the primary processes begin to bifurcate into secondary processes. After 2 days in the differentiation medium (DIV 2), imOLs with three to five primary processes and a sparse arborization predominate this stage. After 4 days of differentiation (DIV 4), mature OLs characterized by multipolar processes and a rich arborization were mainly observed. Note that not all OPCs have the same differentiation speed; the cells shown here represent the majority in each period.
