# Supplementary figures and images for: Dynamic Calcium Release From Endoplasmic Reticulum Mediated by Ryanodine Receptor 3 Is Crucial for Oligodendroglial Differentiation
Source: Front Mol Neurosci. 2018 May 18;11:162. doi: 10.3389/fnmol.2018.00162 (PMC5968115; doi:10.3389/fnmol.2018.00162)

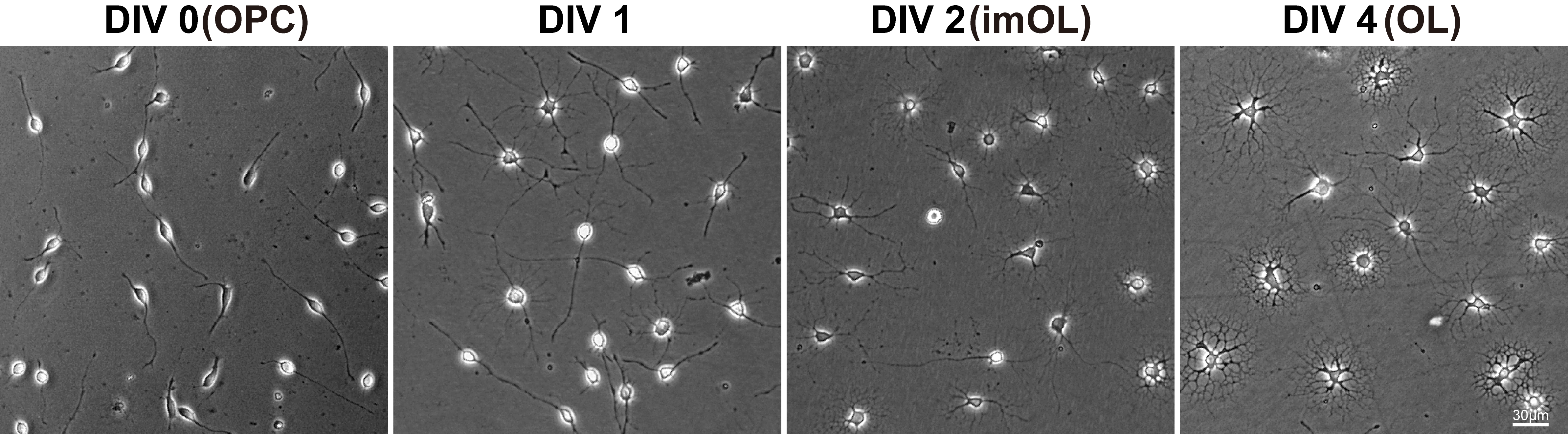

Supplement: Supplementary file 2 [file Image_1.TIF]
